# An advanced deep learning models-based plant disease detection: A review of recent research

**DOI:** 10.3389/fpls.2023.1158933

**Published:** 2023-03-21

**Authors:** Muhammad Shoaib, Babar Shah, Shaker EI-Sappagh, Akhtar Ali, Asad Ullah, Fayadh Alenezi, Tsanko Gechev, Tariq Hussain, Farman Ali

**Affiliations:** ^1^ Department of Computer Science, CECOS University of IT and Emerging Sciences, Peshawar, Pakistan; ^2^ Department of Computer Science and Information Technology, Sarhad University of Science and Information Technology, Peshawar, Pakistan; ^3^ College of Technological Innovation, Zayed University, Dubai, United Arab Emirates; ^4^ Faculty of Computer Science and Engineering, Galala University, Suez, Egypt; ^5^ Information Systems Department, Faculty of Computers and Artificial Intelligence, Benha University, Banha, Egypt; ^6^ Department of Molecular Stress Physiology, Center of Plant Systems Biology and Biotechnology, Plovdiv, Bulgaria; ^7^ Department of Electrical Engineering, College of Engineering, Jouf University, Jouf, Saudi Arabia; ^8^ Department of Plant Physiology and Molecular Biology, University of Plovdiv, Plovdiv, Bulgaria; ^9^ School of Computer Science and Information Engineering, Zhejiang Gongshang University, Hangzhou, China; ^10^ Department of Computer Science and Engineering, School of Convergence, College of Computing and Informatics, Sungkyunkwan University, Seoul, Republic of Korea

**Keywords:** machine learning, deep learning, plant disease detection, image processing, convolutional neural networks, performance evaluation, practical applications

## Abstract

Plants play a crucial role in supplying food globally. Various environmental factors lead to plant diseases which results in significant production losses. However, manual detection of plant diseases is a time-consuming and error-prone process. It can be an unreliable method of identifying and preventing the spread of plant diseases. Adopting advanced technologies such as Machine Learning (ML) and Deep Learning (DL) can help to overcome these challenges by enabling early identification of plant diseases. In this paper, the recent advancements in the use of ML and DL techniques for the identification of plant diseases are explored. The research focuses on publications between 2015 and 2022, and the experiments discussed in this study demonstrate the effectiveness of using these techniques in improving the accuracy and efficiency of plant disease detection. This study also addresses the challenges and limitations associated with using ML and DL for plant disease identification, such as issues with data availability, imaging quality, and the differentiation between healthy and diseased plants. The research provides valuable insights for plant disease detection researchers, practitioners, and industry professionals by offering solutions to these challenges and limitations, providing a comprehensive understanding of the current state of research in this field, highlighting the benefits and limitations of these methods, and proposing potential solutions to overcome the challenges of their implementation.

## 1 Introduction

The use of ML and DL in plant disease detection has gained popularity and shown promising results in accurately identifying plant diseases from digital images. Traditional ML techniques, such as feature extraction and classification, have been widely used in the field of plant disease detection. These methods extract features from images, such as color, texture, and shape, to train a classifier that can differentiate between healthy and diseased plants. These methods have been widely used for the detection of diseases such as leaf blotch, powdery mildew, and rust, as well as disease symptoms from abiotic stresses such as drought and nutrient deficiency (Mohanty et al., 2016; Anjna et al., 2020; Genaev et al., 2021) but have limitations in accurately identifying subtle symptoms of diseases and early-stage disease detection. In addition, they also struggle to process complex and high-resolution images.

Recently, DL techniques such as convolutional neural networks (CNNs) and deep belief networks (DBNs) have been proposed for plant disease detection (Liu et al., 2017; Karthik et al.,2020). These methods involve training a network to learn the underlying features of the images, enabling the identification of subtle symptoms of diseases that traditional image processing methods may not be able to detect (Singh and Misra, 2017; Khan et al., 2021; Liu and Wang, 2021b). DL models can handle complex and large images, making them suitable for high-resolution images (Ullah et al., 2019). However, these methods require a large amount of labeled training data and may not be suitable for unseen diseases. Furthermore, DL models are computationally expensive, which may be a limitation for some applications.

In recent years, several research studies have proposed different ML and DL approaches for plant disease detection. However, most studies have focused on a specific type of disease or a specific plant species. Therefore, more research is needed to develop a generalizable and robust model that can work for different plant species and diseases. Additionally, there is a need for more publicly available datasets for training and evaluating models. One of the recent trends in the field is transfer learning, a technique that allows for reusing pre-trained models on new datasets. Recently, transfer learning and ensemble methods have emerged as popular trends in plant disease detection using ML and DL. Transfer learning involves fine-tuning pre-trained models on a specific dataset to enhance the performance of DL models. Ensemble methods, on the other hand, involve combining multiple models to improve overall performance and reduce dependence on a single model. These approaches have been applied to increase the robustness and accuracy of plant disease detection models. Additionally, it can also prevent overfitting, a common problem in DL models where the model performs well on the training data but poorly on unseen data. Another essential aspect to consider is the use of data augmentation techniques, which is the process of artificially enlarging the size of a dataset by applying random transformations to the images. This approach has been used to increase the diversity of the data and reduce the dependence on a large amount of labeled data.

In conclusion, the application of ML and DL techniques in plant disease detection is a rapidly evolving field with promising results. While these techniques have demonstrated their potential to accurately identify and classify plant diseases. There are still limitations and challenges that need to be addressed. Further research is required to develop generalizable models and make more publicly available datasets for training and evaluation. This review highlights the current state of research in this field and provides a comprehensive understanding of the benefits and limitations of ML and DL techniques for plant disease detection. Its novelty lies in the breadth of coverage of research published from 2015 to 2022, which explores various ML and DL techniques while discussing their advantages, limitations, and potential solutions to overcome implementation challenges. By offering valuable insights into the current state of research in this area, the article is a valuable resource for plant disease detection researchers, practitioners, and industry professionals seeking a thorough understanding of the subject matter.

The following section comprises the contributions of this research article.

- This paper provides an overview of the current developments in the field of plant disease detection using ML and DL techniques. By covering research published between 2015 and 2022, it provides a comprehensive understanding of the state-of-the-art techniques and methodologies used in this field.
- This review examines various ML and DL methods for detecting plant diseases, including image processing, feature extraction, CNNs, and DBNs, and sheds light on the benefits and drawbacks, such as data availability, imaging quality, and differentiation between healthy and diseased plants. The article shows that the use of ML and DL techniques significantly increases the precision and speed of plant disease detection.
- Various datasets related to plant disease detection have been studied in the literature, including PlantVillage, the rice leaf disease dataset, and datasets for insects affecting rice, corn, and soybeans.
- The paper discussed various performance evaluation criteria used to assess the accuracy of plant disease detection models, including the intersection of unions (IoU), dice similarity coefficient (DSC), and accurate recall curves.

The article has seven main sections. A brief overview of plant disease and pest detection and its significance is provided in Section 1. The challenges and issues in the plant disease and pest detection are discussed in Section 2. The deep learning approaches for recognizing images and their applications in plant disease and pest detection are presented in Section 3. The comparison of commonly used datasets and the performance metrics of deep learning methods on different datasets are presented in Section 4. The challenges in existing systems are identified in Section 5. The discussion about the identification of plant diseases and pests is presented in Section 6. Finally, the conclusion of the research work and future research directions are discussed in Section 7.

## 2 Plant disease and pest detection: Challenges and issues

### 2.1 Identifying plant abnormalities and infestations

Artificial Intelligence (AI) technologies have recently been applied to the field of plant pathology for identifying plant abnormalities and infestations. These technologies can have the capability to transform the method in which plant maladies are identified, diagnosed, and managed. In this passage, we will explore the various AI technologies that have been proposed for identifying plant abnormalities and infestations, their advantages and limitations, and the impact of these technologies on the field of plant pathology. One of the most widely used AI technologies in plant pathology is ML. ML algorithms, such as c4.5 classifier, tree bagger, and linear support vector machines, have been applied to the classification of plant diseases from digital images. These algorithms can be trained to recognize specific patterns and symptoms of diseases, making them suitable for the classification of diseases in their primary phases. However, ML algorithms mandate a substantial quantity of data that has been annotated for training and may not be suitable for diseases that have not been seen before.

**Figure 1:**
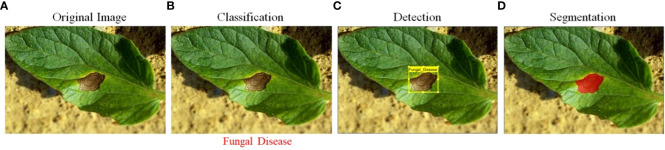
**(A)** Input raw image, **(B)** leaf classification, **(C)** lesion detection, and **(D)** lesion segmentation.

DL technologies, such as CNNs and DBNs, have also been proposed for identifying plant abnormalities and infestations. These technologies have been showing promising outcomes in the detection and identification of lesions from digital images (Kaur and Sharma, 2021; Siddiqua et al., 2022; Wang, 2022). DL models can automatically learn features from the images and can identify subtle symptoms of diseases that traditional image processing methods may not be able to detect. Though, Deep Learning models necessitate a significant volume of labeled training data and involve intensive computational resources, which may be a limitation for some applications. Another AI technology that has been applied to plant pathology is computer vision (CV). CV algorithms, such as object detection and semantic segmentation, can be used to identify and localize specific regions of interest in images, such as plant leaves and symptoms of diseases (Kurmi and Gangwar, 2022; Peng and Wang, 2022). These algorithms can be used to automatically transforming the images into recognizable patterns or characteristics can be integrated with ML or DL algorithms for disease detection and classification. However, CV algorithms need a huge number of labeled image data for model training and may not be suitable for diseases that have not been seen before. **Figure 1** comprises four images, each depicting a different stage of plant disease detection. The first image is the input image, while the next image displays the disease identification results. The third image features lesion detection, and the final image presents the segmentation results of the plant lesion.

AI technologies have shown promising results in identifying plant abnormalities and infestations. ML, DL, and CV based system are utilized for to the classification and lesion segmentation of plant diseases from digital images and could change the method of discovering plant illnesses significantly, diagnosed, and managed (Akbar et al., 2022). However, these technologies need a considerable amount of annotated training data and may not be suitable for diseases that have not been seen before. Further research is needed to develop generalizable models that can be applied to different plant species and diseases, and to make more datasets publicly available for training and evaluating the models. **Table 1** provides comprehensive information about the tools and technologies utilized for plant disease detection. It includes details about the various feature extraction methods, including those based on handcrafted and learning features, as well as the appropriate methods for processing small and large plant image datasets.

**Table 1:** Comparison of different technologies for image processing.

Technology	Core Technique	Necessary Prerequisites	Suitable Contexts
Traditional Image Processing	Manual design of features + classifiers or rules	Significant differentiation between affected and healthy regions, minimal interference, or disturbance.	Plant disease and pest detection in controlled environments
DL	Automatic feature learning using CNNs	Large amounts of appropriate data, high-performance computing units	Adaptation to changes in complex natural environments

### 2.2 Evaluation of conventional techniques for identifying plant diseases and pests

In recent years, ML and DL-based approaches have been increasingly applied to agriculture and botanical studies. These approaches have shown great potential in improving crop yield, identifying plant lesions, and optimizing plant growth. In comparison to traditional approaches, ML and DL-based methods offer several advantages and have the potential to revolutionize the field of agriculture and botanical studies. Traditional approaches in agriculture and botanical studies mainly rely on manual inspection and expert knowledge. These methods are often time-consuming, physically demanding, and susceptible to human mistakes. In contrast, ML and DL-based approaches can automate these tasks, reducing the need for human interference and enhancing precision and efficiency of the process.

ML and DL-based approaches have been used to analyze large amounts of data, including images, sensor data, and weather data, to identify patterns and make predictions. For example, ML algorithms such as c4.5 classifier and tree bagger are being used to predict crop yields, identify plant lesions and pests, and optimize plant growth (Yoosefzadeh-Najafabadi et al., 2021; Cedric et al., 2022; Domingues et al., 2022). DL models, such as CNNs and DBNs, have been applied plant lesion identification based on image analysis and classification, providing better accuracy and robustness compared to traditional image processing methods (Sladojevic et al., 2016; Alzubaidi et al., 2021; Dhaka et al., 2021). The ML and DL-based approaches offer several advantages over traditional methods in agriculture and botanical studies. These methods can automate tasks, increase accuracy and efficiency, and analyze huge quantity of data. Since, these methods require a large size of labeled features and may not be suitable for lesions that have not been seen before. Further research is needed to develop generalizable models that can be applied to different crop species and conditions, and to make more datasets publicly available for predictive model training and model validation for performance analysis.

## 3 Deep learning approaches for recognizing images

DL approaches have become a promising method for detecting plant lesions. These techniques, which are based on RNN have demonstrated success by achieving high accuracy in identifying various plant lesions from images (Xu et al., 2021). By automatically learning features from the images, DL models can accurately identify and classify different disease symptoms, reducing the need for manual feature engineering (Drenkow et al., 2021). Additionally, these models can handle large amounts of data, making them well-suited for large-scale plant lesions detection (Arcaini et al., 2020). Therefore, in review paper, we evaluate the current state-of-the-art in using DL for plant lesions recognition, examining various architectures, techniques, and datasets used in this field. Our aim is to provide a thorough understanding of the current research in this area and identify potential future directions for improving the detection precision and make the identification system more efficient using the DL approaches.

### 3.1 Deep learning theory

Iqbal (Sarker, 2021) popularized the term “Deep Learning” in a 2006 Science article (DL). The article describes a procedure for transforming high-dimensional data into low-dimensional codes using a technique called “autoencoder” networks. These networks are made up of a layers with few parameters that is trained to create vectors of input with high dimensions. The process of fine-tuning the weights of the network can be done using gradient descent, but this method is only effective if the baseline weights are near to a satisfactory solution. The article presents an effective initialization of weights that enables deep autoencoder models to learn the low-dimensional sequences that are more effective than principal component analysis for reducing the dimensionality of data.

DL is a variant of ML that employs multiple-layered AI networks to learn and represent complex patterns in data. It is extensively employed in object recognition, object detection, speech analysis and speech-to-text transcription. In natural language processing, DL-based models are used for tasks such as language translation, text summarization, and sentiment analysis. Additionally, DL is also used in recommendation systems to predict user preferences based on previous actions or interactions. AI vision is a subfield of artificial intelligence concerned with the construction of computers to process and understand the visual contents from the world (Liu et al., 2017).

In traditional manual image classification and recognition methods, the underlying characteristics of an image are extracted through the use of hand-crafted features. These methods, however, are limited in their ability to extract information about the deep and complex characteristics of an image. This is because the manual extraction procedure is extremely reliant on the expertise of an individual conducting the analysis, and can be prone to errors and inconsistencies. Additionally, traditional manual methods are not able to extract information about subtle or hidden features that may be present in an image. In contrast, DL-based image classification and recognition methods use artificial neural networks to automatically extract image features. These methods have been shown to be highly effective in extracting complex and deep features from images, and have been utilized in numerous applications such as object recognition, facial features recognition, and image segmentation. Among the primary benefits of DL-based methods is its capacity to learn features autonomously from input data, rather than relying on manual feature engineering. This allows the model to learn more abstract and subtle features that may be present in the image, leading to improved performance and greater accuracy. Additionally, DL-based methods are also able to handle high-dimensional and complex data, making them particularly well-suited to handling large-scale image datasets. In summary, traditional manual image classification and recognition methods have limitations in extracting deep and complex characteristics of an image, while DL-based methods have been demonstrated greater efficiency and effectiveness in this task by automatically extracting image features, handling high-dimensional and complex data, and learning more abstract and subtle features that may be present in the image (Tran et al., 2015).

DBN (Hasan et al., 2020) is a type of unsupervised DL model that is composed of multiple layers of Restricted Boltzmann Machines (RBMs). Using the plant lesion and pest infestation detection, DBNs have been used to test plant images affected regions to detect various diseases and types of pests, and extract features from images of plant leaves. Studies have shown that DBNs can achieve high accuracy rates in the range of 96-97.5% in classifying images of plant leaves affected by diseases and pests.

Boltzmann’s Deep Machine (DBM) (Salakhutdinov & Larochelle, 2010) is generative stochastic AI model that can be utilized for unsupervised classification to detect the plant lesion. Within the context of conventional plant lesion and pest detection, DBMs have been used to predict labels for images of various plant affected regions by viruses and plant bugs, and extract features from images of plant leaves. Studies have shown that DBMs can achieve high accuracy rates in the range of 96-96.8% in classifying images of plant leaves affected by diseases and pests.

Deep Denoising Autoencoder (Lee et al., 2021) is a variant of autoencoder, which is a neural network architecture that is composed of an encoder module along with a decoder. In the context of traditional plant disease and pest infestation detection, DDA has been used to for two different purposed i.e., noise removal from the plant leaf data and a prediction system to identify plant disease. Studies have shown that DDA can achieve high accuracy rates in the range of 98.3% in classifying images of plant leaves affected by diseases and pests.

Deep CNN (Shoaib et al., 2022a; Shoaib et al., 2022b)is a type of feedforward AI model that is consisting of several hidden layers of convolutional and pooling layers, the CNN model are the best of the DL model for achieving higher detection accuracy using imaging data The CNN model consist of two blocks, the features learning and classification blocks. The features learning block extract various kind of features using the convolutional layer where the features learning is performed at the fully connected layers. The higher accuracy of the CNN model for plant disease classification has proofed to be the best then all other kinds of ML and DL methods. Studies have shown that CNNs can achieve high accuracy rates in the range of 99-99.2% in classifying images of plant leaves affected by diseases and pests.

### 3.2 Convolutional neural network

CNNs are a sort of DL model that are ideally suited for image classification tasks such as leaf disease detection (Zhang et al., 2019; Lin et al., 2020; Stančić et al., 2022). Multiple layers comprise the CNN’s architecture, such as fully connected layers, maxpooling, and normalization layers. The first layer in the CNN is the input layer while the second layer in most of the CNNs is convolutional layers which extract features by applying various kind of 2D filters on the image, the amount of images increase which can then dimensionally reduced pooling also known as down sampling layers, resulting in a more compact representation of the image. Fully connected (FC) layers in a CNN are also known as learnable features, the extracted features are processed in the FC layer for learning and weights optimization. These layers are also responsible for making classification which can be used to recognize various plant diseases. The learning process of CNN model begins with training, the input to the CNN are images along with their labels, after the successful training of the model, the model is able to identify disease types.

**Figure 2:**
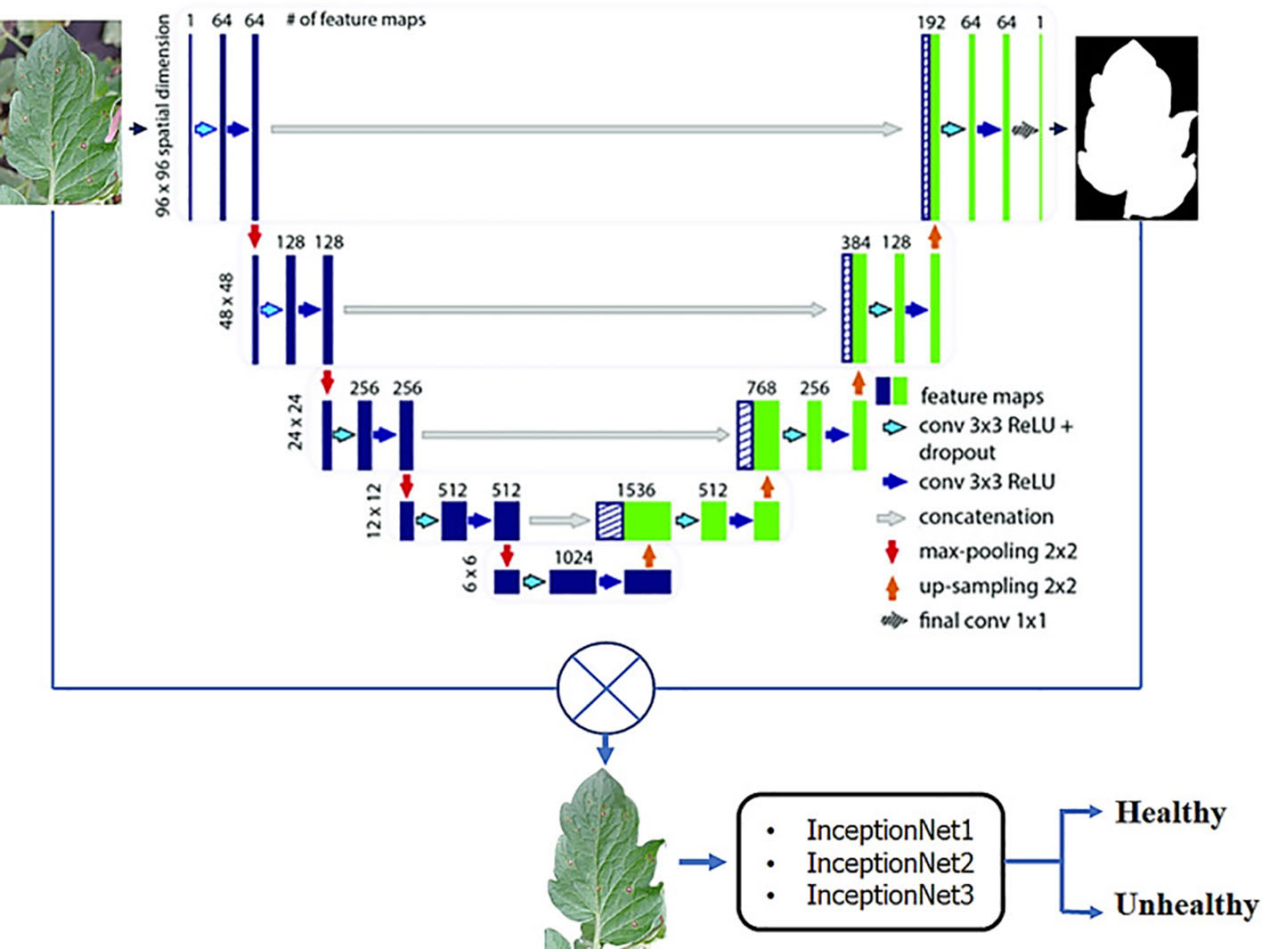
A CNN framework for classifying plants into healthy and unhealthy (Shoaib et al., 2022a).

The decision-making process in a CNN for leaf disease detection starts with the input of an image of a leaf. The image is then passed through the convolutional layers, where features are extracted. The feature vectors are then processed by pooling layers, where the spatial dimensions are reduced. The feature vectors are then transmitted *via* the FC layers, where a decision is made about the presence of a disease or pest. The models output are the probabilities that the leaf is diseased or healthy. CNNs are well-suited for leaf disease detection, thanks to their architecture consisting of up-sampling, down-sampling and learnable layers (Agarwal et al., 2020). The learning process of CNN involves training the network using labeled images of healthy and disease effected plants. **Figure 2** presents a framework for classifying the plants into normal and abnormal plant using leaf data. The framework employs several different Inception architectures, and the final decision is made through a bagging-based approach.

### 3.3 Deep learning using open-source platforms

TensorFlow is a powerful library for dataflow and differentiable programming (Abadi, 2016; Dillon et al., 2017), which allows for efficient computation on a set of devices with powerful hardware’s, that include memory, GPUs and TPUs. Its ability to create dataflow graphs, which describe how data moves through a computation, makes it a popular choice for ML and DL applications. In contrast, Keras is a high-end DL library that operates atop TensorFlow (also some other libraries). It simplifies the creation of DL models by providing a user-friendly API, and it provides a number of pre-built layers and functions, such as convolutional layers and pooling layers, which can be easily added to a model. In recent versions of Tensorflow (2.4 and above). TensorFlow is used to provide low-level operations for building and training models, while Keras is used to provide a higher-level API for building and training models more easily. The use of TensorFlow and Keras together in this research has allowed us to effectively and efficiently solve the problem at hand.

PyTorch is also from the open-source community which has lot of capabilities for developing ML and DL applications (Zhao et al., 2021; Masilamani and Valli, 2021). PyTorch is a powerful library for building and training DL models. It is known for its flexibility and ease of use, making it a popular choice among researchers and practitioners. One of the key features of PyTorch is its dynamic computational graph. Unlike other libraries, such as TensorFlow, which uses a static computational graph, PyTorch allows for the modification of the graph on-the-fly, making it more suitable for research and experimentation. Additionally, PyTorch provides support for distributed training, allowing for efficient training of large models on multiple GPUs. PyTorch also provides a number of pre-built modules, such as convolutional layers and recurrent layers, which can be easily added to a model. This makes it easy to quickly prototype and experiment with different model architectures. Additionally, PyTorch also has a large community that shares pre-trained models, datasets, and tutorials, which helps to make the development process even more efficient.

Caffe (Convolutional Architecture for Fast Feature Embedding) is a Berkeley Vision and Learning Center-developed open-source DL framework (BVLC) and community contributors (Jia et al., 2014). It is a popular choice for image and video classification tasks such as object detection and video summarization, and also consider a good choice for its speed and efficiency in training large models. Caffe is implemented in C++ and has a Python interface, which allows for easy integration with other Python libraries such as NumPy and SciPy. This allows for a high level of flexibility in the design and experimentation of DL models. One of the key features of Caffe is its ability to perform efficient convolutional operations, which are essential for computer vision tasks. Additionally, Caffe supports a wide range of DL models, such as CNN, RNN, Transformers networks. It also provides a number of pre-built layers and functions, such as convolutional layers and pooling layers, which can be easily added to a model (Komar et al., 2018).

The Montreal Institute for Learning Algorithms (MILA) at the University of Montreal created Theano which also covers the open source license and have several packages in the python language for ML and DL (Bahrampour et al., 2015). It is widely used for DL and other numerical computations, and it is known for its ability to optimize and speed up computations on CPUs and GPUs. One of the key features of Theano is its ability to perform symbolic differentiation, which allows for the efficient computation of gradients during the training of DL models (Chung et al., 2017). Additionally, Theano can automatically optimize computations and perform automatic differentiation, which allows for the efficient training of large models. Theano also provides a number of pre-built functions, such as convolutional and recurrent layers, which can be easily added to a model. Theano is implemented in Python, which allows for easy integration with other Python libraries such as NumPy and SciPy. This allows for a high level of flexibility in the design and experimentation of DL models.

**Table 2** in the research article provides a comparison of several popular Artificial Intelligence (AI) frameworks. The table compares the technology, developer, auxiliary devices required, functionality, programming language, and popular applications of each framework. This information is valuable for researchers and practitioners in the field of AI, as it provides an overview of the various options available and the strengths and limitations of each framework. The data presented in **Table 2** can be used to guide the selection of an appropriate AI framework for a specific task or application.

**Table 2:** Comparison of popular artificial intelligence frameworks.

Technology	Developer	Auxiliary Devices	Functionality	Language	Popular Applications
TensorFlow	Google	CPU, GPU, TPU, Mobile	High usability with a large community and extensive documentation	Python	Computer Vision, NLP, Speech Recognition, Robotics, Reinforcement Learning
PyTorch	Facebook	CPU, GPU	High usability for research and development with dynamic computation graphs	Python	Computer Vision, NLP, Speech Recognition, Robotics, Reinforcement Learning
ONNX Runtime	Microsoft	CPU, GPU, TPU, Edge	High usability for deploying models across multiple platforms	Python	Computer Vision, NLP, Speech Recognition, Robotics, Reinforcement Learning
MXNet	Amazon	CPU, GPU, TPU, Mobile	High usability with a variety of language support and performance optimization	Python, C++, R, Scala	Computer Vision, NLP, Speech Recognition, Robotics, Reinforcement Learning
CNTK	Microsoft	CPU, GPU	High usability for large-scale distributed training and models	Python	Computer Vision, NLP, Speech Recognition, Robotics, Reinforcement Learning

### 3.4 Deep learning based plant lesion and pests detection system

This section of the research focuses on the application of DL methods for segmentation plant lesions and pest infestation in botany and agriculture. With the increasing demand for food and the need for sustainable agricultural practices, the prompt identification and handling of illnesses affecting plants and pests is crucial for ensuring crop yields and maintaining the health of crops. DL, with its ability to process large amounts of data and its ability to learn from the data, has proven to be a robust tool for detecting plant diseases and pest infestation. In this section, we present a comprehensive overview of the state-of-the-art DL methods that have been developed for this purpose, including methods for image-based disease and pest detection, as well as methods for data-driven disease and pest detection using sensor data and other types of data. We also discuss the challenges and limitations of these methods and provide insights into future research directions. In particular, we will cover the recent advancements in DL for disease and pest detection, including the use of CNN, recurrent neural networks, and transfer learning techniques. These DL methods have shown to be effective in detecting plant diseases and pest infestation at a high level of accuracy, which can support farmers and agricultural professionals in taking appropriate action to prevent crop losses.

#### 3.4.1 Classification network

Various Convolutional Neural Network (CNN) models which have been utilized to identify plant diseases and pest infestation are discussed. The first model that we will discuss is AlexNet (Antonellis et al., 2015), which is the CNN model developed in 2012. The AlexNet CNN win the classification challenge by achieving the highest accuracy using the 1000 classes Imagenet dataset. AlexNet is known for its high accuracy and speed, and it has been used for a variety of tasks, including plant disease detection. Another popular CNN model is VGG (Soliman et al., 2019), which was established in 2014 by the University of Oxford’s at Visual Geometry Lab. VGG is known for its high accuracy and is often used for image classification tasks. It has been employed to detect plant lesions by extracting hidden patterns from plant leaf data.

ResNet (Szymak et al., 2020), which was developed by Microsoft Research Asia in 2015, is known for its ability to handle very deep networks. It has been used for plant disease detection by using pre-trained ResNet models on the images of the plants. GoogLeNet (Wang et al., 2015), which was developed by Google in 2014, is known for its high accuracy and efficient use of computation resources. It has been used for plant disease detection by fine-tuning pre-trained GoogLeNet models on the images of the plants. InceptionV3, which was developed by Google in 2015, is known for its high accuracy and efficient use of computation resources. It has been used for plant disease detection by fine-tuning pre-trained InceptionV3 models on the images of the plants. DenseNet (Tahir et al., 2022), which was developed in the (Huang et al., 2017), is known for its ability to handle very deep networks and efficient use of computation resources. It has been used for plant disease detection by fine-tuning pre-trained DenseNet models on the images of the plants. These CNN models differ in their architectures, sizes, shapes, and the number of parameters. While AlexNet, VGG, GoogLeNet, InceptionV3, and DenseNet have been widely used for plant disease detection, ResNet is known for its ability to handle very deep networks. All these models have been shown to be effective in detecting plant diseases and pests based on different characteristics such as size, shape, and color, and they can be employed for harvesting characteristics from pictures of the plants which can be used to train a classifier to detect different diseases and pests.

#### 3.4.2 CNN as features descriptor

The article (Sabrol, 2015) “Recent Research on Image Processing and Soft Computing Approaches for Identifying and Categorizing Plant Diseases using CNNs” discusses the use of CNNs for recognizing and classifying plant diseases. The authors review various studies that have used CNNs, which are a type of DL algorithm, to detect and diagnose plant diseases. They also discuss the challenges and limitations of using CNNs, such as the need for large amounts of data, the high computational requirements, and the potential for overfitting. The article concludes by highlighting the potential for further research in this area and the importance of developing accurate and reliable plant disease recognition and classification systems using CNNs.

This research article presents an architecture of Convolutional Neural Networks for determining the variety of crops from image sequences obtained from advanced agro-observation stations (Yalcin and Razavi, 2016). The authors address challenges related to lighting and image quality by implementing preprocessing steps. They then employ the CNN architecture to extract features from the images, highlighting the importance of the construction and depth of the CNN architecture in determining the recognition capability of the network. The accuracy of the model presented is evaluated to perform a comparison between the CNN model with those obtained using a support vector machine (SVM) classifier with the utilization of feature extractors such as Local Binary Patterns (LBP) and Gray-Level Co-Occurrence Matrix. The results of the approach are tested on a dataset collected through a government-supported project in Turkey, which includes over 1,200 agro-stations. The experimental outcomes affirm the efficiency of the suggested technique.

A novel meta-architecture is proposed, which utilizing a CNN designed for distinguishing between healthy and diseased plants (Fuentes et al., 2017b). The authors employed multiple characteristic extractors within the CNN to analyze input images that are divided into their corresponding categories. On the other hand, a CNN-based approach for the identification of various eight classes of rice viruses is presented in (Hasan et al., 2019). The authors performed features extraction using the features learning model and introduced them along with the corresponding labels into a support vector machine (SVM) linear multiclass model for training. The trained model achieved a validation accuracy of 97.5%.

#### 3.4.3 CNN-based predictive systems

In the area of plant illness and pest identification, CNNs have been extensively utilized. One of the first applications of CNNs in this field was the identification of lesions in plant images, utilizing classification networks. The method employed involves training CNNs to recognize specific patterns or features in the input image that are associated with various diseases or pests. After training, the network can be utilized to classify new images as diseased or healthy. The classification of raw images is a straightforward process that utilizes the entire image as input to the CNN. However, this approach may be limited by the presence of irrelevant information or noise in the image, which can negatively impact the performance of the network. In order to address this problem, investigators have proposed utilizing a region of interest (ROI) based approach, in which is the model is taught to categorize specific regions of the image that contain the lesion, rather than the entire image. Multi-category classification is another area of research in this field, which involves training CNNs to recognize multiple types of diseases or pests in the same image. This approach can be more challenging than binary classification, as it requires CNNs to learn more complex and diverse patterns in the input images.

The first broad application of CNNs for plant pest and disease detection was the identification of lesions using categorization networks. Current study issues include the categorization of raw pictures, classification following recognition of regions of interest (ROI), and classification of several categories. Utilizing neural structural models, such as CNN, for direct classification in plant pest identification can be a highly effective strategy. CNN is a DL model that is ideally suited for image classification problems since it can automatically learn picture attributes.

To train the network when the team constructed it independently, a tagged collection of photos of ill and healthy plants was required. There must be a variety of pests and illnesses, plant growth phases, and environmental circumstances within the databases. The team can then construct the network architecture and choose relevant parameters based on the specific features of the intended recipient plant pest and disease. Alternately, during transfer learning, you can employ a CNN model that has already been trained and modify it using data from specific plant pest detection tasks. This method is less computationally intensive and requires less labeled data due to the fact that the pre-trained network has already acquired generic characteristics from huge datasets. Notably, transfer learning enables teams to harness the performance of a model trained in some data that were developed using extensive, varied datasets demonstrated to perform well on similar tasks.

Establishing the weight parameters for multi-objective disease and pest classification networks, obtained through binary learning between healthy and infected samples as well as pests, are uniform. A CNN model is designed that integrates basic metadata and allows training on a single multi-crop model to identify 17 diseases across five cultures by utilizing a unified newly suggested model which has ability to handle multiple crops multi-crop model (Picon et al., 2019). The following goals can be accomplished through the use of the proposed model:

1. Achieve more prosperous and stable shared visual characteristics than a single culture.
2. Is unaffected by diseases that cause similar symptoms across cultures.
3. Seamlessly integrates the context for classifying conditional crop diseases.

Experiments show that the proposed model eliminates 71 percent of classification errors and reduces data imbalance, with a balanced data the proposed model boasts an average accuracy rate of 98%, surpassing the performance of other models.

### 3.5 Identifying lesion locations through neural network analysis

Images are typically processed and labeled using a classification network. However, it is also possible to use a combination of various strategies and methods to determine the location of affected areas and perform pixel-level classification. Some commonly used methods for this purpose include the sliding window approach, the thermal map technique, and the multitasking learning network. These methods involve analyzing the input image and identifying specific regions or areas that correspond to lesions through a systematic and formal analysis process.

The sliding window method is a widely utilized technique for identifying and arranging elements within an image. This method involves moving a small window across the image and analyzing each window using a classification network. This technique is particularly useful for detecting localized features, such as lesions in plant photos, making it a valuable tool. In a study, a CNN classification network incorporating the sliding window method was utilized to develop a system for the identification of plant diseases and pests (Tianjiao et al., 2019). This system incorporates ML, feature fusion, identification, and location regression estimation through the use of sliding window technology. The software demonstrated an ability to identify 70-82% of 29 typical symptoms when used in the field.

The graphic illustrates a temperature chart that illustrates the importance of various regions within an image. The darker the hue, the greater the importance of that region. Specifically, darker tones on the heat map indicate a higher likelihood of lesion detection in plants affected by diseases and pests. In a study conducted by (Dechant et al., 2017), a convolutional neural network (CNN) was trained to generate thermal maps of corn disease images, which were then used to classify the entire image as infected or non-infected. The process of creating a thermal map for a single image takes approximately 2 minutes and requires 2 GB of memory. Identifying a group of three thermal cards for execution, on the other hand, takes less than a second and requires 600 bytes of memory. The results of the study showed that the test data set had an accuracy rate of 98.7%. In a separate study, (Wiesner-hanks et al., 2019) used the thermal map system to accurately identify contour zones for maize diseases with a 96.22% accuracy rate in 2019. This method of detection is highly precise and can identify lesions as small as a few millimeters, making it the most advanced method of aerial plant disease detection to date.

A multitasking learning network is a network that is capable of both categorizing and segmenting plant afflictions and pests. Unlike a pure predictive model, which is only able to categorize images at the image level, multitasking networks add a branch that can accurately locate the affected region of plant diseases. This is achieved by sharing the results of characteristic extraction between the two branches. As a result, the multitasking learning network uses a detection hierarchy to generate precise lesion detection results, which reduces the sampling requirements for the classification network. In a study by (Shougang et al., 2020), a VGCNN model followed by deconvolution (DGVGCNN) was developed to detect afflictions of plant leaves resulting from shadows, obstructions, and luminosity levels. The implementation of deconvolution redirects the CNN classifier’s attention to the precise locations of the afflictions, resulting in a highly robust model with a disease class identification accuracy of 97.81%, a lesion segmentation pixel accuracy of 96.44%, and a disease class recognition accuracy of 98.15%.

**Figure 3:**
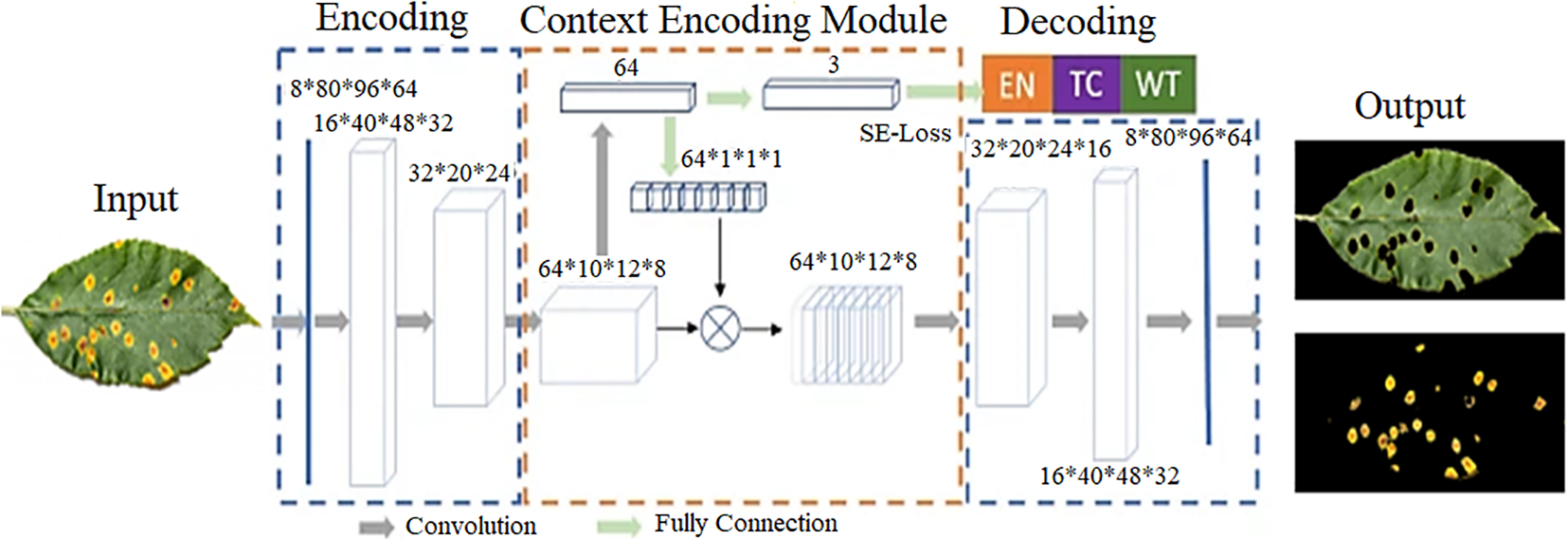
CANet neural network-based disease detection and ROI segmentation (Shoaib et al., 2022b).

**Figure 3** presents architecture of the CANet neural network. utilized for plant lesion detection and segmentation. The figure provides a visual representation of the various components and structure of the network, such as the input layer, intermediate hidden layers, and the final output layer. This information is valuable for researchers and practitioners who are interested in understanding the underlying mechanics of the CANet network and how it performs lesion detection and segmentation.

**Table 3** provides a comparison of the pros and cons of various object detection and classification methods for identifying diseases in the leaves of plants. The table compares five methods including Convolutional Neural Networks (CNNs), Transfer learning with CNNs, Multitasking learning networks, Deconvolution-guided VGNet (DGVGNet), and traditional methods such as manual inspection and microscopy. This information is valuable for researchers and practitioners in the area of identifying plant lesions, as it provides a comprehensive comparison of the strengths and limitations of each method, enabling them to make informed decisions about which method is most suitable for their needs. The data presented in **Table 3** can act as a guide for future studies and development in the field of plant disease detection.

The research community as a whole has come to acknowledge the utility of taxonomic network systems for the detection of plant pests, and a significant amount of study and investigation is currently being carried out in this field. **Table 3** offers a full comparison of the several sub-methods that make up the categorized network system, showing the benefits and drawbacks of each option (Mohanty et al., 2016; Brahimi et al., 2018; Garcia and Barbedo, 2019). It is essential to keep in mind that the method that will prove to be the most effective will change depending on the particular use case as well as the resources. It should also be mentioned that while this table does illustrate the performance of each approach, it should not be considered to be an exhaustive comparison because the results may differ depending on the particular data sets and environmental conditions that are used.

**Table 3:** Comparison of pros and cons of various object detection and classification methods for plant leaf disease detection.

Method	Advantages	Disadvantages
(CNNs)	High accuracy, able to detect small lesions	Need large amounts of labeled data for training
Transfer learning with CNNs	Improved performance using pre-trained models	Limited to the specific task and dataset the pre-trained model was trained on
Multitasking learning networks	Can classify and segment simultaneously, reducing sampling requirements for classification	Complex architecture requires more computational resources
Deconvolution-guided VGNet (DGVGNet)	Robust in occlusion, low light, and other conditions, with high accuracy	Requires specific architecture and computationally intensive
Traditional methods (e.g. manual inspection, microscopy)	Low-cost and widely available	Time-consuming, prone to human error and subjectivity

#### 3.5.1 Object detection networks for plant lesion detection

Object localization is a fundamental task in computer vision and is closely associated with the traditional detection of plant pests. The objective of this task is to acquire knowledge about the location of objects and their corresponding categories. In recent years, various algorithms for object detection based on DL have been developed. These include single-stage networks such as SSD (W. Liu et al., 2016) and YOLO (Dumitrescu et al., 2022; Peng and Wang, 2022; Shoaib and Sayed, 2022), as well as a networks with multi-stages, like YOLOv1 (Nasirahmadi et al., 2021). These techniques are commonly employed in the identification of plant lesions and pests. The single-stage network makes use of network features to directly forecast the site and classification of blemishes, whereas the two-stage network first generates a candidate box (proposal) with lesions before proceeding to the object detection process.

#### 3.5.2 Pest and plant lesion localization using multi-stage network

Faster R-CNN is a two-part object detection system that uses a common feature extractor to obtain a map of features from an input image. The network then utilizes a Region Proposal Network (RPN) to calculate anchor box confidences and generate proposals. The features maps of the proposed regions are then connected to the ROI pooling layer to enhance the initial detection results and finally determine the location and type of the lesion. This method improves upon traditional structures by incorporating modifications to the feature extractor, anchor ratios, ROI pooling, and loss functions that are tailored to the specific characteristics of plant disease and pest infestation detection. In a study conducted by (Fuentes et al., 2017a), the Faster R-CNN was used for the first time to accurately locate tomato diseases and pests infestation in a dataset containing 4800 images of 11 different categories. When using deep feature extractors like VGG-Net and ResNet, the mean average precision (mAP) value was calculated 88.66%.

**Figure 4:**
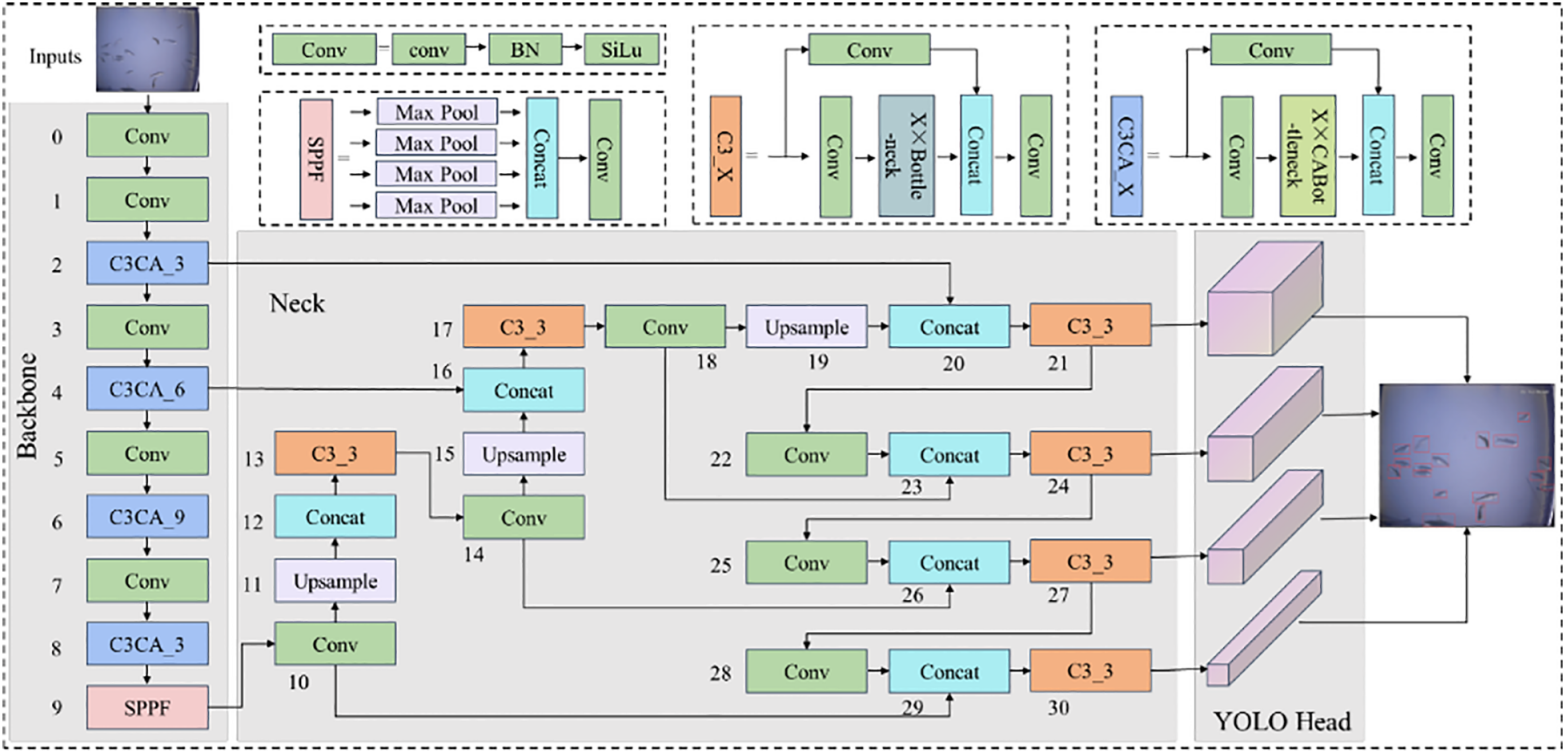
YOLOv5 architecture (Li et al., 2022).

The YOLOv5 architecture is visually represented in **Figure 4**, which depicts its structure and organization. The network comprises three primary components: the input layer, the hidden layers, and the output layer. The input layer is where data is initially fed into the network for processing. The hidden layers are responsible for executing complex computations and transformations on the input data, and their performance plays a critical role in determining the network’s accuracy. The output layer generates final predictions by outputting the bounding boxes and class probabilities for objects detected in the input image. The figure provides detailed labels and annotations to explain how the network’s components interact. This visual representation helps researchers and developers gain a better understanding of the network’s mechanics and identify areas for performance enhancement. Overall, **Figure 4** is an essential tool for anyone seeking to deepen their understanding of the YOLOv5 architecture. In 2019, (Liu and Wang, 2021b) a modification was suggested for the Faster R-CNN framework to automatically detect beet spot lesion by altering the parameters of the CNN model. A total of 142 images were used for testing and validation, resulting in an overall correct ranking rate of 96.84%. (Zhou et al., 2019) a rapid detection system for rice diseases was proposed by integrating the FCM-Kmeans and YOLOv2 algorithms. The system showed a detection accuracy of 97.33% with a processing time of 0.18s for rice blast, 93.24% accuracy and 0.22s processing time for bacterial blight, and 97.75% accuracy and 0.32s processing time for sheath burn, based on the evaluation of 3010 images. (Xie et al., 2020) proposed the DR-IACNN model based on the faster mechanism to ensure efficiency, a custom dataset is developed that contains the vine leaf lesions (GLDD), and the Faster R-CNN detector employe of a Inception-v2 architecture, the Inception-ResNetv2 architecture. The proposed model showed a mean average precision (mAP) accuracy of 83.7% and a detection rate of 12.09 frames per second. The two-stage detection network was designed to improve the real-time performance and practicality of the detection system. However, it still lacks in terms of speed compared to the speed of one-stage detection model.

#### 3.5.3 One-stage network based plant lesion detection

In recent years, object detection has become an essential tool for diagnosing plant afflictions and pests. YOLO (You Only Look Once) is one of the most widely used object detection techniques. It is a real-time, single-pass object detector that utilizes a single CNN to predict the category and position of objects in an image. Variations of the YOLO algorithm, such as YOLOv2 and YOLOv3, and other various methods have been developed to enhance the accuracy of object recognition while maintaining real-time performance. Another popular object detection technique is SSD (Single Shot MultiBox Detector), which similarly to YOLO, uses a single CNN to predict the type and position of objects in an image. However, SSD makes predictions about the size of objects based on multiple feature maps that are scaled differently, making it better suited for identifying small objects with greater precision than YOLO.

Faster R-CNN is a two-stage object detection system that generates a set of potential object regions using a Region Proposal Network (RPN), and then uses a separate CNN to classify and locate objects within these proposals. Despite being slower than YOLO and SSD, Faster R-CNN has been shown to achieve a higher level of accuracy. When it comes to detecting plant diseases and pests, YOLO, SSD, and Faster R-CNN are all commonly used methods. The choice of algorithm will depend on the specific requirements of the application, such as accuracy, speed, and memory consumption. For real-time applications that prioritize speed, YOLO may be the best option, but for applications that require a higher level of accuracy, SSD and Faster R-CNN may be more suitable.

In this study (Singh et al., 2020), the authors explore the potential of utilizing computer vision techniques for the early and widespread detection of plant diseases. To aid in this effort, a custom dataset, named PlantDoc, was developed for visual plant disease identification. The dataset includes 3,451 data points across 12 plant species and 14 disease categories and was created through a combination of web scraping and human annotation, requiring 352 hours of effort. To demonstrate the effectiveness of the dataset, three plant disease classification models were trained and results showed an improvement in accuracy of up to 29%. The authors believe that this dataset can serve as a valuable resource in the implementation of computer vision methods for plant disease detection.

(Zhang et al., 2019) proposed a novel approach to the detection of small agricultural pests by combining an improved version of the YOLOv3 algorithm with a spatial pyramid pooling technique. This method addresses the issue of low recognition accuracy caused by the variable posture and scale of crop pests by applying deconvolution, combining oversampling and convolution operations. This approach allows for the detection of small samples of pests in an image, thus enhancing the accuracy of the detection. The method was evaluated using 20 different groups of pests collected in real-world conditions, resulting in an average identification accuracy of 88.07%. In recent years, many studies have employed detection networks to classify pathogens and pests (Fuentes et al., 2017a). It is expected that in the future, more advanced detection models will be utilized for the identification of plant maladies and infestations, as object segmentation networks in computer vision continue to evolve.

In recent times, the detection of plant maladies and infestations has increasingly relied upon the use of two-stage models, which prioritize accuracy. However, there is a growing trend towards the use of single-stage models, which prioritize speed. There has been debate over whether detection networks can replace classification networks in this field. The primary goal of a segmentation network is first to identify the presence of plant maladies and infestations, whereas the goal of a predictive model based on a classification scheme is to categorize these diseases and pests. It is important to note that the visual recognition network provides information on the specific category of diseases and pests that need to be identified. To accurately locate areas of plant disease and pest infestation, detailed annotation is necessary. From this perspective, it may seem that the detection network includes the steps of the classification network. However, it is important to remember that the predetermined categories of plant diseases and pests do not always align with actual results. While the detection network may provide accurate results in different patterns, these patterns may not accurately represent the individuality of specific plant maladies and infestations, and may only indicate the presence of certain kinds of illness and bugs in a specific area. In such cases, the use of a classification network may be necessary. In conclusion, both classification networks and detection networks are important for efficient plant disease and pest detection, but classification networks have more capabilities than detection networks.

### 3.6 Deep learning-based segmentation network

The segmentation network transforms the task of detecting plant and pest diseases into semantic segmentation, which includes separating lesions from healthy areas. By dividing the lesion’s area in half, it calculates the position, rank, and associated geometric properties (including length, width, surface, contour, center, etc.). Fully convolutional networks include the R-CNN mask (Lin et al., 2020) and completely convolutional networks (FCNs) (Shelhamer et al., 2017).

#### 3.6.1 Fully connected neural network

A complete convolution neural network is used to segment the image’s semantics (FCN). FCN uses convolution to extract and encode the input image features, then deconvolution or oversampling to gradually restore the characteristic image to its original size. FCN is used in almost all semantic segmentation models today. Traditional plant and pest disease segmentation methods are categorized as conventional FCN, U-net (Navab et al., 2015), and SegNet (Badrinarayanan et al., 2017) according to variations in the architecture of the FCN network.

A proposed technique for the segmentation of maize leaf disease employs a fully convolutional neural network (FCN)(Wang and Zhang, 2018). The process begins with preprocessing and enhancing the captured image data, followed by the creation of training and test sets for DL. The centralized image is then input into the FCN, where feature maps are generated through multiple layers of convolution, pooling, and activation. The feature map is then up sampled to match the dimensions of the input image. The final step is the restoration of the segmented image’s resolution through the process of deconvolution, resulting in the output of the segmentation process. This method was applied to segment common maize leaf disease images and it was found that the segmentation effect was satisfactory with an accuracy rate exceeding 98%.

The proposed approach employs an improved fully convolutional network (FCN) to precisely segment point regions from crop leaf images with complicated backgrounds (Wang et al., 2019). The strategy addresses the difficulty of reliably identifying sick spots in complicated field situations. The training method of the proposed system employs a collection of crop leaf pictures with healthy and sick sections. The algorithm’s performance is tested using measures such as accuracy and intersectional union ratio (IoU) to determine its ability to effectively partition lesion regions from pictures. The experimental findings demonstrate that the algorithm segments the spot area in complicated backdrop crop leaf images with great precision.

U-Net is a popular CNN architecture for image segmentation tasks. The architecture is named U-Net because it is U-shaped, with encoder and decoder sections connected by a bottleneck (Shoaib et al., 2022a). The encoder section of the network consists of a series of convolutional and clustering layers that extract entities from the input image. These features then pass through the bottleneck, where they are up sampled and connected to the feature map from the encoder. This allows the network to use both superficial and fundamental image attributes when making predictions. The decoder part of the network then uses these connected feature maps to generate the final segmentation map. The U-Net architecture is particularly useful for image segmentation tasks because it is able to handle class imbalance problems, where some areas of the image contain more target objects than others.

This paper proposes a semantic segmentation model that uses CNNs to recognize and segment powdery mildew in individual pixel-level images of cucumber lea (Lin et al., 2019). The suggested model obtains an average pixel accuracy of 97.12%, a joint intersection ratio score of 79.54%, and a dice accuracy of 81.54% based on 20 test samples. These results demonstrate that the proposed model outperforms established segmentation techniques such as the gaussian mixture model, random forests, and fuzzy c means. Overall, the proposed model can accurately detect powdery mildew on cucumber leaves at the pixel level, making it a valuable tool for cucumber breeders to assess the severity of powdery mildew.

A novel approach to detect vineyard mildew is proposed, which utilizes DL segmentation on Unmanned Aerial Vehicle (UAV) images (Kerkech et al., 2022). The method involves combining visible and infrared images from two different sensors and using a newly developed image registration technique to align and fuse the information from the two sensors. A fully convolutional neural network is then applied to classify each pixel into different categories, such as shadow, ground, healthy, or symptom. The proposed method achieved an impressive detection rate of 89% at the vine level and 84% at the leaf level, indicating its potential for computer-aided disease detection in vineyards.

#### 3.6.2 Mask regional-CNN

Mask R-CNN is an effective DL model that is perfect for plant pest detection. It is an extension of the Faster R-CNN model and can recognize objects and segment instances (Permanasari et al., 2022). The primary advantage of Mask R-CNN over other models such as YOLO and SSD is its capacity to produce object masks that allow more precise image object location. This is especially beneficial for detecting plant pests, as it enables for more precise identification of afflicted areas. In addition, Mask R-CNN is able to handle overlapping object instances, which is a common issue in plant pest detection due to the presence of several instances of the same pest and disease in a single image. This makes the Mask R-CNN a highly adaptable model that is appropriate for a variety of plant pest identification applications.

In this study (Stewart et al., 2019), an R-CNN based on a masking scheme was utilized to segregate foci of northern plant leaf spots in UAV-captured pictures. The model is trained with a specific data set that recognizes and segments individual lesions in the test set with precision. The average intersectional union ratio (IOU) between the ground reality and the projected lesions was 79.31%, and the average accuracy was 97.24% at a threshold of 60% IOU. In addition, the average accuracy when the IOU threshold ranged from 55% to 90% was 65%. This study illustrates the potential of combining drone technology with advanced instance segmentation techniques based on DL to offer precise, high-throughput quantitative measures of plant diseases.

Using deep CNNs and object detection models, the authors of this paper offer two strategies for tomato disease detection (Wang et al., 2019). These techniques employ two distinct techniques, YOLO and SSD. The YOLO detector is used to categorize tomato disease kinds, while the SSD model is used to classify and separate the ROI-contaminated areas on tomato leaves. Four distinct deep CNNs are merged with two object detection models in order to obtain the optimal model for tomato disease detection. A dataset is generated from the Internet and then split for experimental purposes into training sets, validation sets, and test sets. The experimental findings demonstrate that the proposed approach can accurately and effectively identify eleven tomato diseases and segment contaminated leaf areas.

## 4 Comparing datasets and evaluating performance

This section starts by providing an overview of the evaluation metrics for DL models, specifically focusing on those that pertain to plant disease and pest detection. It then delves into the various datasets that are relevant to this field, and subsequently, conducts a thorough analysis of the recent DL models that have been proposed for the detection of plant diseases and pests.

### 4.1 Evaluating plant disease detection using benchmark datasets

**Figure 5:**
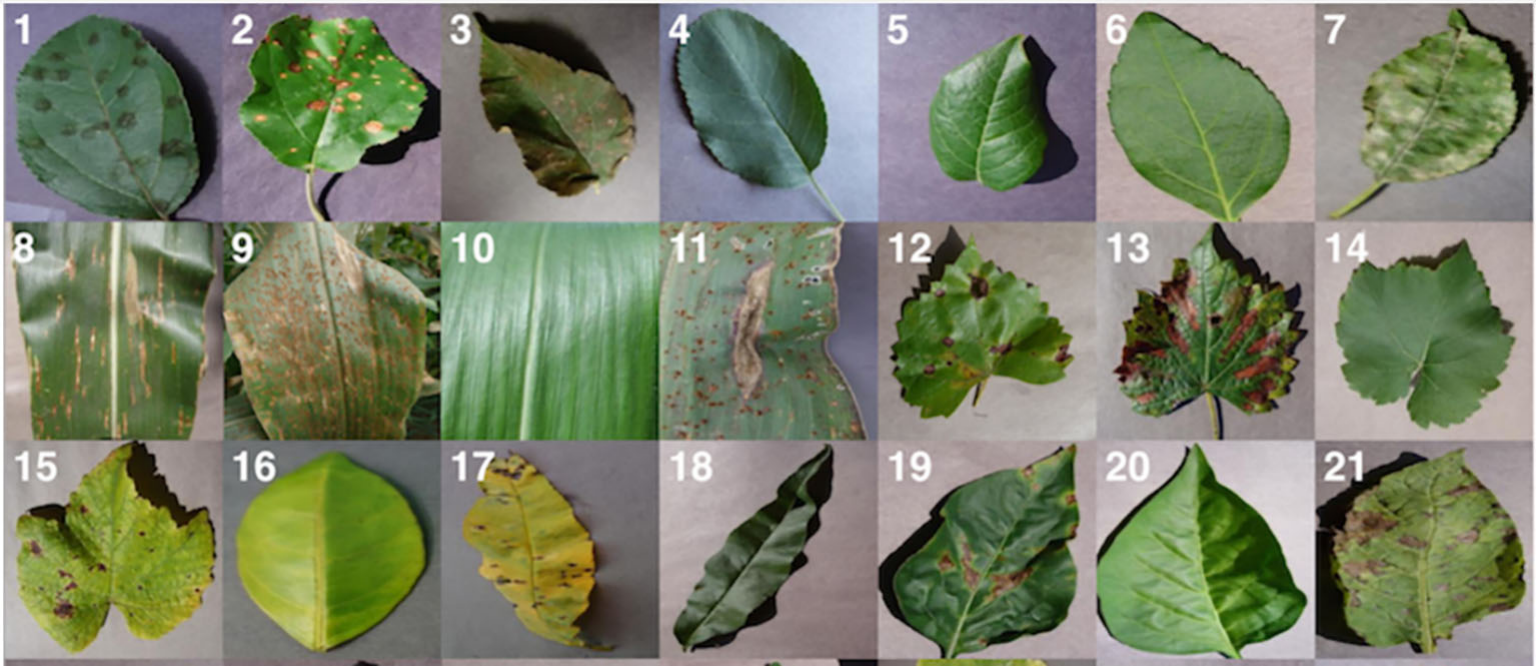
Some random images from plantvillage dataset (Hughes and Salathé, 2015).

The PlantVillage dataset is a compilation of crop photos with labels indicating the presence of various illnesses (Hughes and Salathé, 2015). It features 38,000 photos of 14 distinct crops, including, among others, tomatoes, potatoes, and peppers. The photographs were gathered from many sources, including public databases, research institutions, and individual contributors. The dataset is divided into a training set, a validation set, and a test set, with the training set including the majority of the photos. The scientific community uses this dataset extensively to develop and evaluate DL models for plant disease detection. **Figure 5** showcases a selection of images obtained from the PlantVillage dataset, which is a comprehensive dataset containing thousands of images of various plant species. These images depict a wide range of plant conditions, such as healthy plants, plants affected by pests, and plants afflicted by various diseases, which enables researchers and practitioners to gain a comprehensive understanding of the variability in plant growth and development. Moreover, the diverse range of plant species represented in this figure provides an in-depth and realistic representation of the variability in plant types. The images included in this figure capture the nuanced differences in plant morphology, such as leaf shape, color, and texture, which can be useful for developing and validating deep learning models for plant disease detection. The AgriVision collection (Chiu et al., 2020), which contains photos of numerous crops and their diseases, and the Plant Disease Identification dataset, which contains photographs of damaged and healthy plant leaves, are two other significant datasets.

**Figure 6:**
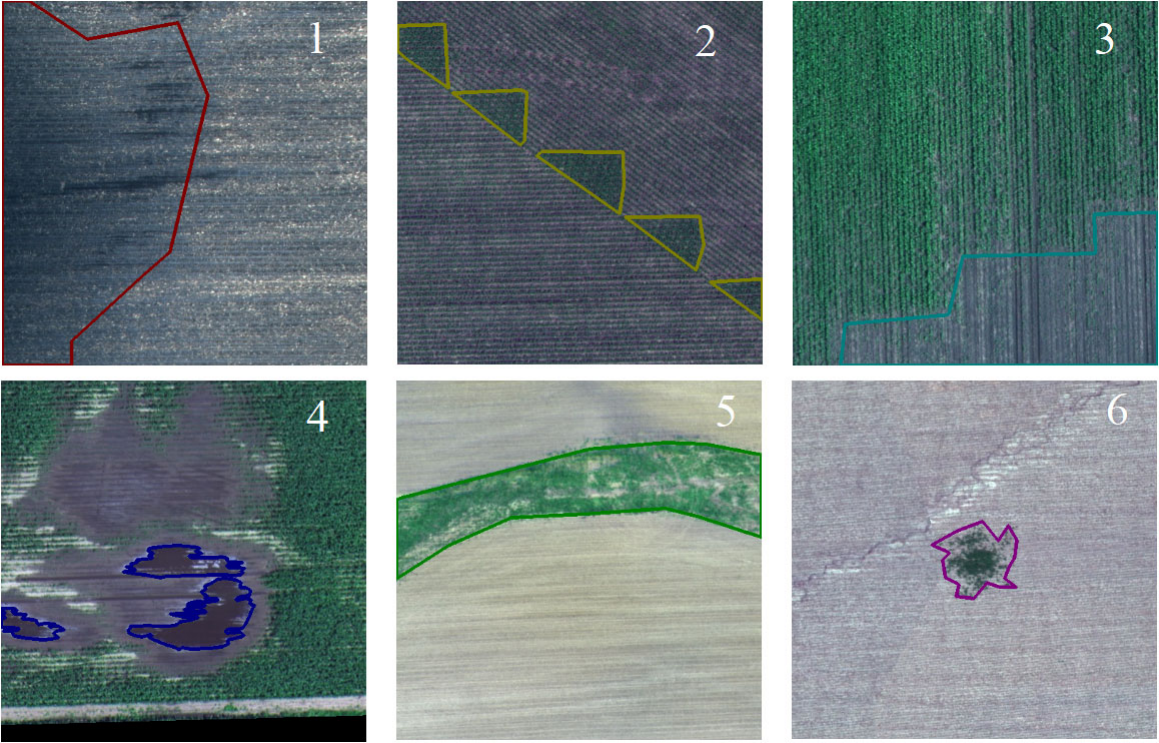
Some random images from agri-vision dataset (Chiu et al., 2020).

**Figure 6** showcases a selection of random images obtained from the Agri-Vision dataset. These images depict various crops and their growth conditions, including both healthy and diseased plants. This figure serves as a visual representation of the types of data available in the Agri-Vision dataset, providing insight into the range and diversity of data contained within the dataset. The Crop Disease dataset comprises photos of 14 crops affected by 27 diseases, whereas the Plant-Pathology-2020 dataset provides images of plant leaves damaged by 38 diseases. All of these datasets are widely utilized by the research community and contribute to the creation and evaluation of DL models for plant disease detection.

**Table 4** provides a summary of benchmark datasets commonly used for plant disease and pest detection. The table includes information on the name of the dataset, a brief description, the type of data contained within the dataset, and the types of diseases and pests covered. This information is valuable for researchers and practitioners who are looking to evaluate or compare their algorithms or models against existing datasets.

**Table 4:** Plant disease and pest detection from benchmark datasets.

Dataset Name	Description	Type of Data	Disease/Pest Types Covered
PlantVillage	A publicly available dataset of over 54,000 images of diseased and healthy plant leaves, compiled from experts and citizen scientists	RGB Images	38 crop species and 38 disease types
PlantClef	A dataset of over 9,000 images of plant leaves, used for the annual PlantCLEF benchmarking campaign	RGB Images	Multiple crop species and disease types
Open Plant Disease Dataset	A dataset of over 8,000 images of plant leaves, compiled from various sources including university research and citizen scientists	RGB and infrared images	Multiple crop species and disease types
Plant Disease Detection in Cotton Images	A dataset of over 5,000 images of cotton leaves, compiled by the National Cotton Council of America for disease detection research	RGB Images	Cotton leaf diseases
AGRONOMI-Net	A dataset of over 3,000 images of various crops, compiled by the AGRONOMI-Net project for disease detection research	RGB and thermal images	Multiple crop species and disease types
Northern Leaf Blight (NLB) Lesions	A dataset of images of corn plants affected by NLB collected from a field environment	RGB	Northern Leaf Blight Disease
Insects from rice, maize, soybean	A dataset of images of insects on rice, maize, and soybean plants collected from a field environment	RGB	Rice Planthoppers, Brown Planthoppers, and Whiteflies
Pest and Disease Image Database (PDID)	A dataset of over 7,000 images of diseased and healthy plants collected from a field environment	RGB	Various crop species and diseases
Plant Disease and Pest Recognition (PDPR)	A dataset of over 30,000 images of diseased and healthy plants collected from a field environment	RGB	Various crop species and diseases

### 4.2 Evaluation indices

**Figure 7:**
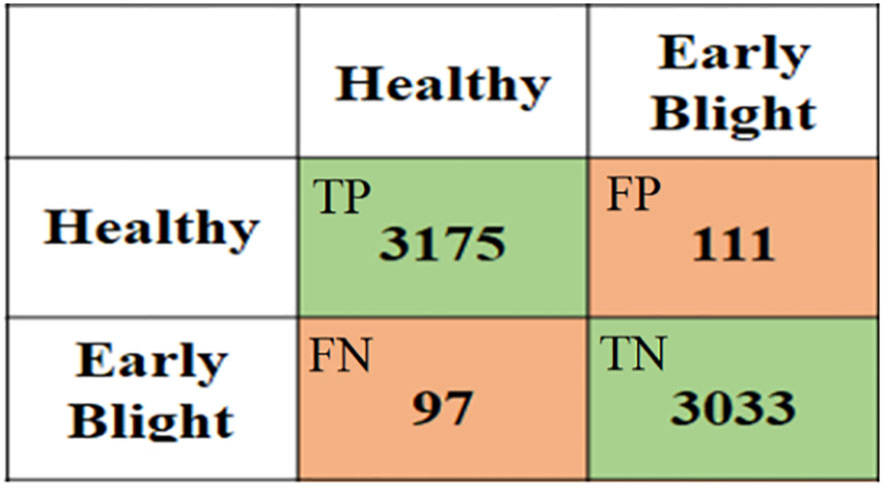
An example of a confusion matrix where the rows show the predicted results while columns represent actual classes.

There are several performance metrics commonly used for evaluating the performance of plant disease classification, detection, and segmentation models. **Figure 7** displays an example of a confusion matrix, a widely used evaluation metric in machine learning. The matrix represents the results of a classification algorithm, where each row represents the predicted class of a given sample and each column represents the actual class of that sample. The entries in the matrix show the number of samples that have been correctly or incorrectly classified. By examining the entries in the confusion matrix, it is possible to gain insight into the performance of the classification algorithm and identify areas for improvement.

**Accuracy:** This is the proportion of correctly classified instances out of the total number of instances. Mathematically, it is represented as:


$$Accurracy=\;\frac{True\;Positive\;Ratio+True\;Negative\;Ratio}{Total\;number\;of\;Samples}$$

**Precision:** This is the proportion of correctly classified positive instances out of the total number of predicted positive instances. Mathematically, it is represented as:


$$Precision=\;\frac{True\;Positive\;Ratio}{True\;Positive\;Ratio+False\;Positive\;Ratio}$$

**Recall (Sensitivity):** This is the proportion of correctly classified positive instances out of the total number of actual positive instances. Mathematically, it is represented as:


$$Recall=\;\frac{True\;Positive\;Ratio}{True\;Positive\;Ratio+False\;Negative\;Ratio}$$

**F1 Score:** This is the harmonic mean of precision and recall. Mathematically, it is represented as:


$$F1-Score=\frac{2*\left(Precision*Recall\right)}{Precision+Recall}$$

**Intersection over Union (IoU):** This is used to evaluate the performance of segmentation models. It is the ratio of the area of intersection of the predicted segmentation and the ground truth segmentation to the area of the union of the two. Mathematically, it is represented as:


$$IoU=\;\frac{True\;Positive\;Ration}{True\;Positive\;Ration+False\;Positive\;Ration+False\;Negative\;Ratio}$$

**Dice coefficient:** This is another metric used for evaluating segmentation performance. It is a measure of the similarity between the predicted segmentation and the ground truth segmentation, and it ranges from 0 to 1. Mathematically, it is represented as:


$$Dice\;Coefficient=\;\frac{2*TP}{2*TP+FP+FN}$$

**Jaccard index:** This is another metric used for evaluating segmentation performance. It is the ratio of the area of intersection of the predicted segmentation and the ground truth segmentation to the area of the union of the two. Mathematically, it is represented as:


$$Jaccard\;Index=\frac{TP}{TP+FP+FN}$$

**Figure 8:**
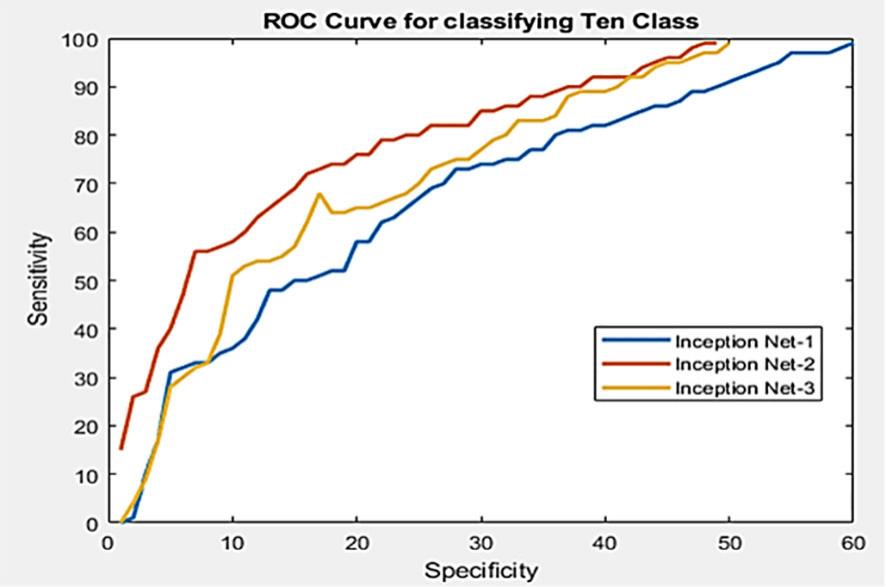
An example of performance comparison between three models using the ROC curve (Shoaib et al., 2022a).

**Receiver Operating Characteristic:** This curve is a graphical representation of the performance of a binary classifier system. **Figure 8** presents an example of a performance comparison between three models using a receiver operating characteristic (ROC) curve. The ROC curve is a widely used evaluation metric in machine learning that graphically summarizes the performance of a binary classifier by plotting the true positive rate against the false positive rate for different classification thresholds. The ROC curve provides a visual representation of the trade-off between the false positive rate and true positive rate, allowing practitioners to compare the performance of different models at different operating points. It plots the true positive rate (TPR) against the false positive rate (FPR) at various threshold settings. The TPR, also known as the sensitivity, recall or hit rate, is the number of true positive predictions divided by the number of actual positive cases. The FPR, also known as the fall-out or probability of false alarm, is the number of false positive predictions divided by the number of actual negative cases. The ROC curve can be mathematically represented as TPR = (TP)/(TP + FN) and FPR = (FP)/(FP + TN), where TP, FP, TN, and FN are true positives, false positives, true negatives, and false negatives, respectively. The area under the ROC curve (AUC) is a measure of the classifier’s performance, with a value of 1 indicating perfect performance and a value of 0.5 indicating no better than random.

**Area Under the Curve:** This AUC is also a performance measure used to evaluate the performance of the binary classifier. It is derived by integrating the true positive rate (TPR) relative to the false positive rate (FPR) overall thresholds. TPR is determined by dividing the number of true positives by the total number of true positive instances (TP + FN), whereas FPR is determined by dividing the number of false positives by the total number of true negative cases (FP + TN). AUC goes from 0 to 1, where 1 corresponds to a perfect classifier and 0.5 corresponds to a random classifier. A greater AUC value suggests superior classification ability.

### 4.3 Performance comparison of existing algorithms

This article examines in depth the most recent developments in DL-based plant pest identification. The papers examined in this article, published between 2015 and 2022, focus on the detection, classification, and segmentation of plant pests and lesions using ML and DL approaches. This research employs several methodologies, including image processing, feature extraction, and classifier creation. In addition, DL models, namely CNNs, have been widely applied to accurately detect and categorize plant illnesses. This article addresses the problems and limits of utilizing ML and DL algorithms for plant lesions identification, including data availability, image quality, and subtle differences between healthy and diseased plants. This paper also examines the current state of practical applications of ML and DL techniques in plant abnormal region detection and provides viable solutions to address the obstacles and limits of these technologies.

The research covered in this article indicates that the employment of ML and DL approaches enhances the accuracy and efficiency of plant lesion detection greatly. The most prevalent evaluation criteria are mean accuracy (mAP), F1 score, and frames per second (FPS). However, a gap still exists between the intricacy of the images of infectious maladies and infestations utilized in this study and the usage of mobile devices to identify pest and lesions infestations in the field in real-time. This paper is a valuable resource for plant lesions detection researchers, practitioners, and industry experts. It provides a comprehensive understanding of the current state of research utilizing ML and DL techniques for plant lesions detection, highlights the benefits and limitations of these methods, and proposes potential solutions to overcome the challenges of their implementation. In addition, the need for larger and more intricate experimental data sets was identified as a subject for further investigation.

## 5 Challenges in existing systems

### 5.1 Overcoming small dataset challenge

Using data augmentation techniques to fictitiously expand the dataset is one method. Another strategy is to use knowledge from models that have already been trained on bigger data sets to smaller data sets. The third approach successfully addresses the small sample problem by combining the first two approaches. Despite these achievements, a significant obstacle in the field of DL-based plant pest identification is still the limited dataset problem. Future research should therefore concentrate on creating new tools and techniques to successfully address this issue and enhance the functionality of DL models in this domain.

### 5.2 Plant image amplification for lesions segmentation

In recent years, data amplification technology has been utilized extensively in the field of plant pest detection in order to circumvent the issue of small data set size. These techniques involve the use of image manipulation operations including mirroring, translation, shearing, scaling, and contrast alteration in order to create additional training examples for a DL model. In order to enrich tiny datasets, generative adversarial networks (GANs) (Goodfellow et al., 2020) and automated encoders (Pu et al., 2016) were also utilized to generate fresh, diverse samples. It has been demonstrated that these strategies considerably enhance the performance of DL models for plant pest detection. It is essential to emphasize, however, that the efficacy of these strategies is contingent on the quality and diversity of the original dataset. Additionally, the produced samples must be thoroughly analyzed to confirm their suitability for DL model training. Data amplification, synthesis, and generative approaches are crucial components of plant pest detection model training using DL.

### 5.3 Transfer learning for plant disease and pest detection

Transfer learning is a technique that applies models that have been trained on large, generic datasets to more specific tasks with fewer data. This method is especially beneficial in the field of plant pest detection, where annotated data is frequently sparse. Pretrained models can be customized for specific localized plant pest and abnormality detection tasks by refining parameters or fine-tuning certain components. Transfer learning can increase model performance and minimize model development expenses, according to studies. For example, (Oppenheim et al., 2019) used the VGG network to recognize natural light images of contaminated potatoes of various sizes, colors, and forms. (Too et al., 2019) discovered that as the number of iterations grew, the accuracy of dense nets improved when employing fine and contrast parameters. In addition, (Chen et al., 2020) demonstrate that transfer learning can accurately diagnose rice lesions photos in complicated situations with an average accuracy of 94 percent, exceeding standard training.

### 5.4 Optimizing network structure for plant lesion segmentation

A properly designed array structure can greatly minimize the number of samples required for plant pest and lesions segmentation. Utilizing several color channels, merging depth-separate convolution, and adding starting structures are some of the strategies employed by researchers to increase feature extraction. Specifically, Identification of plant leaf diseases using RGB pictures and a convolutional neural network with three channels (TCCNN) in (Zhang et al., 2019). An enhanced CNN approach that uses deep separable convolution to detect illnesses in grapevine leaves is proposed in (Liu et al., 2020), with 94.35% accuracy and faster convergence than classic ResNet and GoogLeNet structures. These examples illustrate the significance of examining network patterns for detecting plant pests and diseases with limited sample numbers.

### 5.5 Small-size lesions in early identification

The primary role of the attention mechanism is to pinpoint the area of interest and swiftly discard unnecessary data. A weighted sum approach with weighted coefficients can be used to separate the features and reduce background noise in plant and pest images by analyzing the images’ features. Specifically, the Attention Mechanism module can build a new noise reduction fusion function using the Softmax function by capturing the prominent image, isolating the item from the context, and utilizing and fusing the feature image with the original feature image. The attention mechanism can efficiently choose data and assign enhanced resources to the ROIs, allowing for additional precise identification of minor lesions during the early stages of pest infestations and diseases. Numerous research, such as (Karthik et al., 2020) have demonstrated the efficacy of the attention based prediction system. On the industrial village dataset, the network residual attention mechanism was evaluated with an overall accuracy of 98%. In addition, to improve the precision of tiny lesion detection, research can concentrate on creating more robust preprocessing algorithms to reduce background noise and enhance picture resolution. This may involve techniques such as picture enhancement, image denoising, and image super-resolution.

### 5.6 Fine-grained identification

The identification of plant diseases and pests is a challenging task that is often made more complex by variations in the visual characteristics of affected plants. These variations can be attributed to external factors such as uneven lighting, extensive occlusion, and fuzzy details (Wang et al., 2017). Furthermore, variations in the presence of illness and the growth of a pest can lead to subtle differences in the characterization of the same diseases and pests in different regions, resulting in “intra-class distinctions” (Barbedo, 2018). Additionally, there is a problem of “inter-class resemblance,” which arises from similarities in the biological morphology and lifestyles of subclasses of diseases and pests, making it difficult for plant pathologists to differentiate between them.

In actual agricultural settings, the presence of background disturbances might make it harder to detect plant pests and diseases (Garcia and Barbedo, 2018). Environment complexity and interactions with other items can further complicate the detecting procedure. It is essential to highlight, however, that images obtained under controlled conditions may not truly depict the difficulties of spotting pests and illnesses in their natural habitats. Despite advancements in DL techniques, identifying pests and diseases in real-world contexts remains a technological issue with accuracy and robustness constraints. Current research focuses mostly on the fine-grained identification of individual pest populations, and it is challenging to apply these methods to mobile, intelligent agricultural equipment for large-scale identification. Therefore, additional study is required to address these obstacles and enhance the effectiveness of agricultural decision management.

### 5.7 Low and high illumination problem

In the past, researchers captured photos of plant pests and illnesses using indoor lightboxes (Martinelli et al., 2015). Despite the fact that this method efficiently eliminates the impacts of outdoor lighting, hence simplifying picture processing, it is essential to remember that photographs captured under natural lighting circumstances might vary significantly. The dynamic nature of natural light and the limited range of the camera’s dynamic light source might create color distortion if the camera settings are not appropriately adjusted. Moreover, the visual attributes of plant illnesses and infestations may be impacted by factors such as viewing angle and distance, offering a formidable challenge to visual recognition algorithms. This emphasizes the significance of addressing light conditions and image capture techniques when researching pests and plant diseases, as these factors can significantly impact the accuracy and dependability of results.

### 5.8 Challenges posed by obstruction

Currently, the majority of scientists tend to concentrate on detecting plant pests and diseases in particular ecosystems, rather than addressing the setting as a whole. Frequently, they directly intercept areas of interest in the gathered photos without completely resolving the occlusion issue. This results in low recognition accuracy and restricted applicability. There are numerous types of occlusions, including differences in leaf location, branches, external lighting, and hybrid designs. These occlusion issues are ubiquitous in the natural environment, where a lack of distinguishing characteristics and overlapping noise makes it difficult to identify plant pests and illnesses. In addition, varying degrees of occlusion may have varying effects on the recognition process, leading to errors or missed detections. Some researchers have found it challenging to identify plant pests and diseases under extreme conditions, such as in the shadow, despite recent breakthroughs in DL algorithms (Liu and Wang, 2020; Liu and Wang, 2021a). However, in recent years, a solid foundation has been established for plant utilization and pest identification in actual situations.

To improve the performance of plant pest and disease detection, it is necessary to increase the originality and efficiency of the underlying architecture, which must be improved for optimal results of lightweight network topologies. The difficulty of constructing a core framework is frequently reliant on the performance of the hardware system. Consequently, optimizing the underlying framework is crucial for enhancing efficiency and performance. Moreover, processing blockage might be unanticipated and difficult to anticipate. Therefore, it is essential to lower the complexity of model formation while simultaneously enhancing GAN exploration and preserving detection precision. GANs have the capacity to manage postural shifts and turbulent settings well. However, GAN architecture is still in its infancy and prone to issues during the learning and training phase. To aid in the evaluation of the model’s efficacy, it is essential to do additional research on the network’s outcomes.

### 5.9 Challenges in detection efficiency

DL algorithms have proven more effective than conventional approaches, although they are computationally intensive. This causes slower inspections and challenges in satisfying real-time requirements, particularly when a high level of detection precision is required. Frequently, in order to resolve this issue, it is required to minimize the amount of data used, which might result in poor planning and erroneous or lost identification. Therefore, it is vital to create an accurate and effective algorithm for threat identification. In agricultural applications, the process of detecting pests and illnesses using DL approaches requires three main steps: data labeling, model training, and model inference. The model inference is particularly applicable to agricultural applications in real-time. However, it should be highlighted that the majority of current mechanisms for disease and bug detection in plants rely on accurate identification, while less emphasis has been paid to the dependability of model inference. For instance, the author of (Kc et al., 2019) employs an ensemble convolutional structural framework to identify plant foliar diseases in order to improve the efficiency of the model calculation process and satisfy real agricultural needs. This approach was compared to various different models, and the decreased MobileNet classification accuracy was 92.12%, with parameters that were 31 times lower than VGG and 6 times lower than MobileNet. This demonstrates that real-time crop disease diagnostics on mobile devices with limited resources strike a solid balance between speed and accuracy.

## 6 Discussion

### 6.1 Datasets for identifying plant diseases and pests

The advancement of DL technology has greatly contributed to the improvement of Identifying and managing infestations in crops and plants. Theoretical developments in image identification mechanisms have paved the way for identifying complex diseases and pests. However, it should be noted that the majority of research in this field is limited to laboratory studies and relies heavily on photographs of plant diseases and pests that have been collected. Previous research often focused on identifying specific features such as disease spots, insect appearance, and leaf identification. However, it is important to consider that plant growth is cyclical, consistent, seasonal, and regional in nature. Therefore, it is crucial to gather sample images from various stages of plant growth, different seasons, and regions to ensure a more comprehensive understanding of plant diseases and pests. This will improve the robustness and generalization of the model.

It is essential to keep in mind that the properties of plant diseases or insects which may vary in various phases of crop development. Moreover, photos of different plant species may change by location. Consequently, the majority of current research findings may not be universally relevant. Even if the recognition rate of a single test is high, the reliability of data collected at other times or locations cannot be confirmed. Much of the present study has concentrated on images in the visible spectrum, but it is crucial to remember that electromagnetic waves generate vast amounts of data outside of the visible spectrum. It is necessary to merge data from multiple sources, such as visible, near-infrared, and multispectral, to generate a comprehensive dataset on plant diseases. Future studies will emphasize the use of multi-dimensional concatenation (fusion) techniques to gather and recognize information on plant insects. It should also be highlighted that a database containing photographs of many wild plant pests and illnesses is currently in the process of being compiled. Future studies can use wearable automatic field spore traps, drone aerial photography systems, agricultural Internet of Things monitoring devices, etc. to identify wide regions of farmland, compensating for the absence of randomness in prior studies’ image samples. Improve the overall performance of the algorithm by ensuring the dataset is complete and accurate.

### 6.2 Pre-emptive detection of plant diseases and pests

Early Identifying the various forms of plant diseases and pests can be a difficult task. due to the fact that symptoms are not always apparent, either through visual inspection or computer analysis. In terms of research and necessity, however, early identification is essential since it helps prevent and control the spread and growth of pests and diseases. Recording photographs under favorable lighting conditions, such as sunny weather, enhance image quality, but capturing images on overcast days complicates preprocessing and decreases identification accuracy. In addition, it might be difficult to understand even high-resolution photos during the first phases of plant pests and diseases. It is necessary to incorporate meteorological and plant health data, such as temperature and humidity, to efficiently identify and predict pests and diseases. Rarely has this technique been utilized to diagnose early plant pests and diseases.

### 6.3 Neural network learning and development

Manual pest and disease testing are tough since it is difficult to sample for all pests and diseases, and oftentimes only accurate data are available (positive samples). However, the majority of existing systems for plant pest and disease identification utilizing DL are based on supervised learning, which involves the time-consuming collection of huge labeled datasets. Consequently, it is worthwhile to research methods of unsupervised learning. In addition, DL can be a “black box” with little explanatory power, necessitating the labeling of many learning samples for end-to-end learning. In order to assist training and network learning, it may be advantageous to combine past knowledge of brain-like computers with human visual cognitive models.

However, depth models demand a great deal of memory and testing time, making them inappropriate for mobile platforms with limited resources. Therefore, it is necessary to find solutions to reduce model complexity and speed without sacrificing precision. Choosing appropriate hyperparameters, such as learning rate, filter size, step size, and number, has proven to be a significant challenge when applying DL models to new tasks. These hyperparameters have high internal dependencies, so even small changes can have a substantial effect on the final training results.

### 6.4 Cross-disciplinary study

Theories such as scientific evidence and agronomic plant defenses will be merged to produce more effective field diagnostic models for crop growth and disease identification. Using this technology, plant and pest diseases can be diagnosed with greater speed and precision. In the future, it will be important to shift beyond simple surface image analysis to determine the underlying mechanisms by which pests and diseases occur, together with a full understanding of crop growth patterns, environmental conditions, and other pertinent elements. DL approaches have been demonstrated to address complicated problems that regular image processing and ML methods cannot. Despite the fact that the practical implementation of this technology is still in its infancy, it has enormous development and application potential. To reach this potential, specialists from a variety of fields, such as agriculture and plant protection, must combine their knowledge and experience with DL algorithms and models. In addition, the outcomes of this study will need to be incorporated into agricultural gear and equipment to accomplish the desired theoretical effect.

### 6.5 Deep learning for plant stress phenotyping: Trends and perspectives

DL and ML technologies are successful in detecting and analyzing lesions from severe abiotic stresses, such as drought. In the past decade, global crop production losses due to drought have totaled approximately $30 billion (Agarwal et al., 2020). In 2012, a severe drought impacted 80% of agricultural land in the US, resulting in over two-thirds of counties being declared disaster areas. According to FAO (UN) reports, drought is the primary cause of agricultural production loss. Drought stress causes 34% of crop and livestock production loss in LDCs and LMICs, costing 37 billion USD. Agriculture sustains 82% of all drought impact. Understanding how plants adapt to stress, especially drought, is essential for securing crop yields in agriculture. DL and ML approaches are therefore a major advance in the field of plant stress biology. ML and DL can be used to categorize plant stress phenotyping problems into four categories: identification, classification, quantification, and prediction (Singh et al., 2020). These categories represent a progression from simple feature extraction to increasingly more complex information extraction from images. Identification involves detecting specific stress types, such as sudden death syndrome in soybeans or rust in wheat. Classification uses ML to categorize the images based on stress symptoms and signatures, dividing the visual data into distinct stress classes, such as low, medium, or high stress categories. The final category, prediction, involves anticipating plant stress before visible symptoms appear, providing a timely and cost-effective way to control stress and advancing precision and prescriptive agriculture.

### 6.6 Limitations of this study

The study presented in this paper has some limitations that are attributed to its research methodology. Firstly, the study’s scope is confined to publications from 2015 to 2022, implying that recent developments in plant disease detection may not be covered. Moreover, the review does not encompass an all-inclusive list of Machine Learning (ML) and Deep Learning (DL) techniques for plant disease detection. Nevertheless, the study provides an overview of the most commonly used techniques, their advantages, limitations, and probable solutions to overcome implementation challenges. Finally, the study fails to include an extensive examination of the economic and environmental impacts of ML and DL techniques on plant disease detection. Hence, additional research is necessary to scrutinize the potential benefits and disadvantages of these techniques regarding production losses and resource utilization.

### 6.7 Practical implications of study

The practical implications of our research include:

- Improved plant disease detection: Our research highlights the effectiveness of using ML and DL techniques for plant disease detection, which can help improve the accuracy and efficiency of disease detection compared to traditional manual methods. By adopting these advanced technologies, farmers and plant disease specialists can detect diseases at an early stage, preventing further spread and reducing the risk of crop losses.
- Development of generalizable models: Our research emphasizes the need for developing generalizable models that can work for different plant species and diseases. The development of such models can save time and effort for researchers and practitioners, making it easier to detect and classify plant diseases in various settings.
- Accessible datasets for training and evaluation: The research emphasizes the need for more publicly available datasets for training and evaluating ML and DL models for plant disease detection. The availability of such datasets can help researchers and practitioners develop more accurate and robust models, enhancing the performance of disease detection systems.
- Potential for cost reduction: The use of ML and DL techniques in plant disease detection can reduce the need for manual labor and the cost of plant disease detection. This can be especially useful for farmers and small-scale agricultural operations who may not have access to expensive equipment or specialized expertise.
- Transferable knowledge to other fields: Our research also has the potential to inform research and development in other fields, such as medical imaging and remote sensing. The techniques and methodologies used in plant disease detection can be applied to other fields, providing insights into the potential applications of ML and DL in various domains.

## 7 Conclusions

The DL and ML technologies have greatly improved the detection and management of crop and plant infestations. Advances in image recognition have made it possible to identify complicated diseases and pests. However, most research in this area is limited to lab-based studies and heavily relies on collected plant disease and pest photos. To enhance the robustness and generalization of the model, it’s important to gather images from various plant growth stages, seasons, and regions. Early identification of plant diseases and pests is crucial in preventing and controlling their spread and growth, thus incorporating meteorological and plant health data, such as temperature and humidity, is necessary for efficient identification and prediction. Unsupervised learning and integrating past knowledge of brain-like computers with human visual cognition can aid in DL model training and network learning. Achieving the full potential of this technology requires collaboration between specialists from agriculture and plant protection, combining their knowledge and experience with DL algorithms and models, and integrating the results into farming equipment. The paper explores the recent progress in using ML and DL techniques for plant disease identification, based on publications from 2015 to 2022. It demonstrates the benefits of these techniques in increasing the accuracy and efficiency of disease detection, but also acknowledges the challenges, such as data availability, imaging quality, and distinguishing healthy from diseased plants. The study finds that the use of DL and ML has significantly improved the ability to identify and detect plant diseases. The novelty of this research lies in its comprehensive analysis of the recent developments in using ML and DL techniques for plant disease identification, along with proposed solutions to address the challenges and limitations associated with their implementation. By exploring the benefits and drawbacks of various methods, and offering valuable insights for researchers and industry professionals, this study contributes to the advancement of plant disease detection and prevention.

## Authors contributions

MS, BS, SE-S, AA, AU, FayA, TG, TH, and FarA performed the data analysis, conceptualized this study, designed the experimental plan, conducted experiments, wrote the original draft, revised the manuscript. All authors contributed to the article and approved the submitted version.
