# Corrigendum: An advanced deep learning models-based plant disease detection: a review of recent research

**DOI:** 10.3389/fpls.2023.1282443

**Published:** 2023-09-27

**Authors:** Muhammad Shoaib, Babar Shah, Shaker EI-Sappagh, Akhtar Ali, Asad Ullah, Fayadh Alenezi, Tsanko Gechev, Tariq Hussain, Farman Ali

**Affiliations:** ^1^ Department of Computer Science, CECOS University of IT and Emerging Sciences, Peshawar, Pakistan; ^2^ Department of Computer Science and Information Technology, Sarhad University of Science and Information Technology, Peshawar, Pakistan; ^3^ College of Technological Innovation, Zayed University, Dubai, United Arab Emirates; ^4^ Faculty of Computer Science and Engineering, Galala University, Suez, Egypt; ^5^ Information Systems Department, Faculty of Computers and Artificial Intelligence, Benha University, Banha, Egypt; ^6^ Department of Molecular Stress Physiology, Center of Plant Systems Biology and Biotechnology, Plovdiv, Bulgaria; ^7^ Department of Electrical Engineering, College of Engineering, Jouf University, Jouf, Saudi Arabia; ^8^ Department of Plant Physiology and Molecular Biology, University of Plovdiv, Plovdiv, Bulgaria; ^9^ School of Computer Science and Information Engineering, Zhejiang Gongshang University, Hangzhou, China; ^10^ Department of Computer Science and Engineering, School of Convergence, College of Computing and Informatics, Sungkyunkwan University, Seoul, Republic of Korea

**Keywords:** machine learning, deep learning, plant disease detection, image processing, convolutional neural networks, performance evaluation, practical applications

## Incorrect affiliation

In the published article, there was an error in **affiliation 10**.

Instead of “Department of Software, Sejong University, Seoul, Republic of Korea”, it should be “Department of Computer Science and Engineering, School of Convergence, College of Computing and Informatics, Sungkyunkwan University, Seoul, Republic of Korea”.

The authors apologize for this error and state that this does not change the scientific conclusions of the article in any way. The original article has been updated.

